# Delay-Oriented Roadside Unit Deployment for Highway Intersections in Vehicular Ad Hoc Networks

**DOI:** 10.3390/s24134377

**Published:** 2024-07-05

**Authors:** Gan Luan, Zhenjia Chen, Chunyi Yue, Shi Guan

**Affiliations:** 1 School of Information and Communication Engineering, Hainan University, Haikou 570228, China; 2 Hainan Engineering Research Center of Marine Electromagnetic Spectrum, Haikou 570228, China

**Keywords:** delay, deployment, highway, intersection, roadside unit, vehicular ad hoc network, vehicle-to-infrastructure communication

## Abstract

Optimizing the deployment of roadside units (RSUs) holds great potential for enhancing the delay performance of vehicular ad hoc networks. However, there has been limited focus on devising RSU deployment strategies tailored specifically for highway intersections. In this study, we introduce a novel probabilistic model to characterize events occurring around highway intersections. By leveraging this model, we analytically determine the expected event reporting delays for both highway segments and intersections. Subsequently, we propose an RSU deployment scheme specifically designed for highway intersections, aimed at minimizing the expected event reporting delay. To implement this scheme, we introduce an innovative algorithm named cooperative walking. Through illustrative examples, we demonstrate that our proposed RSU deployment strategy for highway intersections outperforms the commonly employed uniform RSU deployment scheme and the previously proposed balloon method in terms of delay performance.

## 1. Introduction

Highway intersections, characterized by intricate designs and high vehicle speeds, are prone to a significant number of traffic accidents [1]. The geometric features of highway intersections affect the severity of accidents [2]. Efficient vehicular communication is crucial at highway intersections for disseminating traffic information, aiding in accident prevention, and facilitating driving decision-making. With the evolution of intelligent transportation systems (ITS), vehicular ad hoc networks (VANETs) have become pivotal in facilitating wireless communications for safety applications [3]. Additionally, roadside units (RSUs) play a vital role in ensuring the sustainability of VANETs [4]. RSUs boast robust communication, computing, and caching capabilities, serving as pivotal components for facilitating wireless communication, data collection, dissemination, and providing vehicles with Internet access [5]. By ensuring seamless connectivity and minimizing latency, RSUs support a wide array of applications spanning traffic management, navigation assistance, autonomous driving, and entertainment services. However, the deployment of RSUs poses numerous challenges, including the optimization of their location, number, and configuration, while balancing cost-effectiveness and coordinating communication with vehicles. Additionally, RSUs must adapt to the dynamic and heterogeneous nature of vehicular networks.

In response to these challenges, researchers have proposed various methods and models to address the complexities of RSU deployment [5]. These approaches encompass various strategies, such as delay-oriented schemes, coverage maximization approaches, and cost-effective methods for placing RSUs. Various traffic scenarios are explored in these studies, spanning urban, rural, and highway settings. Some research specifically targets improving connectivity and performance at urban intersections, where the complexities of traffic flow dynamics and communication requirements are particularly intricate. These efforts contribute to the realization of safer, more efficient, and interconnected road networks that cater to the evolving needs of modern transportation. However, RSU deployment schemes designed specifically for highway intersections are scarce, and those designed for urban intersections are often ill-suited for highway systems.

Highway intersections and urban intersections exhibit notable disparities [6]. First, in terms of vehicle speeds and traffic densities, highway intersections accommodate high-speed vehicles with substantial traffic volume, necessitating ample space and ramps for convenient entry and exit. Conversely, urban intersections experience lower vehicle speeds and denser traffic, prompting the incorporation of pedestrian safety features like zebra crossings, signal lights, and signs. Second, regarding design and layouts, highway intersections often employ stack interchange designs, integrating ramps, overpasses, and underpasses to facilitate seamless vehicle direction changes without obstructing traffic flow. On the other hand, urban intersections are typically at-grade, with intricate designs catering to pedestrians, cyclists, and public transportation needs. Lastly, in terms of land area occupied, highway intersections require more significant land allocation due to design intricacies, locations, and the need for extensive ramps and infrastructure. In contrast, urban intersections occupy less land but necessitate careful planning to accommodate diverse traffic demands and ensure safety. Consequently, while RSUs are usually only allocated to critical intersections in urban settings, highway deployments necessitate strategic RSU positioning along both intersections and road segments. Hence, a novel RSU deployment scheme tailored for highway intersections is imperative.

When an event or accident occurs, timely reporting to the ITS within half of the golden period is crucial, as highlighted in [7]. However, the primary cause of delay in event reporting lies in transmitting messages to RSUs. Therefore, it is imperative to carefully calculate RSU locations, especially considering different road designs, to ensure timely event reporting to the ITS. This strategic placement of RSUs is essential for minimizing reporting delays and maximizing the system’s efficiency in managing traffic incidents. In our paper, we introduce an RSU deployment scheme specifically designed for highway intersections. Our approach calculates the optimal RSU locations along intersection road segments by minimizing event reporting delays. The innovations of our work can be summarized as follows:

1. We propose a probabilistic model that combines the probability density functions (PDFs) of event locations along the road segments of a highway intersection to describe the overall distribution of event locations in the vicinity.
2. Analytical results for PDFs of event reporting delays and their expectations are derived for both highway road segments and intersections with the deployment of multiple RSUs.
3. We have developed a novel RSU deployment scheme tailored for highway intersections, with the primary objective of minimizing the expected event reporting time to nearby RSUs. This innovative approach simultaneously addresses delay variations across different road segments, accommodating distinct traffic intensities, vehicle speeds, and event location probability distributions. By doing so, we achieve an overarching optimal RSU deployment strategy for the highway intersection. Moreover, this scheme is highly adaptable and can be seamlessly extended to tackle the RSU deployment challenges across an entire highway system encompassing multiple intersections.
4. We introduce the cooperative walking algorithm as a means to implement our RSU deployment scheme for highway intersections. This innovative algorithm provides a creative approach to identifying optimal RSU locations for both highway segments and intersections, thereby circumventing the need for exhaustive mathematical calculations.

The rest of this paper is structured as follows: Section 2 presents related work. Section 3 introduces the system model. Analytical results and the proposed delay-oriented RSU deployment scheme are derived in Section 4. Our cooperative walking algorithm, implementing the scheme, is illustrated in Section 5. Example results are discussed in Section 6, followed by the conclusion in Section 7.

## 2. Related Work

### 2.1. RSU Deployment in Urban Environments

Considerable attention has been focused on RSU deployment in urban environments, where the optimization of coverage within VANETs is of paramount importance [5]. In [8], the authors introduced GeoCover, an RSU deployment scheme aimed at maximizing VANET coverage in urban areas. GeoCover incorporates geometric road network features, vehicle mobility patterns, service quality requirements, and resource constraints in its derivation. To address these considerations, the authors utilize genetic algorithms and greedy algorithms. However, the reliance on fixed hotspot information in this study, which is dynamic in real-world scenarios, introduces vulnerability into the results.

In [9], a strategy for RSU deployment aims to balance communication efficiency and traffic coverage. The authors use an optimization model to deploy RSUs considering traffic demands, reducing data transmission delay and maximizing vehicles served. They employ numerical simulation and optimization theory, verifying the method’s effectiveness on a 4 km × 4 km virtual road network. Initially, they define a data transmission delay model, considering vehicle arrivals and communication range limitations. Then, they propose a node-to-node delay model based on traffic demands and vehicle density. They formulate a multi-objective optimization problem, reducing VANETs delay and increasing vehicles served by RSUs. Using a genetic algorithm, they find that deploying few RSUs significantly improves VANETs efficiency, serving most vehicles. Particularly, when RSUs cover approximately 25% of road segments, there is a notable reduction in network delay and service to over 70% of vehicles. However, employing a genetic algorithm may require significant time to find the optimal solution, and results may be influenced by the initial population setting.

Abdrabou and Zhuang [10] contribute an analytical framework to investigate the multi-hop delay of packets under disrupted connectivity, leading to the derivation of a maximum inter-RSU distance based on their findings. However, the lack of consideration for network dynamics restricts its applicability in VANETs.

### 2.2. RSU Deployment for Urban Road Intersections

Additional research delves into RSU placement algorithms specifically tailored for urban road intersections, as discussed in the following papers. In [11], the authors introduce an RSU deployment algorithm designed for urban scenario intersections, leveraging factors such as vehicle density, intersection popularity, and intersection particularity to determine RSU allocation. The article introduces three RSU deployment algorithms: greedy, dynamic, and hybrid. Greedy prioritizes intersections for RSU placement, dynamic ensures uniform RSU distribution and minimizes coverage overlap, while hybrid combines both approaches. Using real urban data from Seoul’s JungGu/JongroGu, YongsanGu, and GangnamGu areas, the study found similar results for evenly distributed intersections like GangnamGu. However, in areas with complex, uneven intersections like YongsanGu and JungGu/JongroGu, the dynamic algorithm performed best in reducing RSUs and overlap, while the hybrid algorithm balanced between greedy and dynamic. Further verification across varied urban layouts, traffic patterns, and cultures is needed. Additionally, the study may not fully consider factors like traffic flow variability and accident rates when determining intersection priority. This approach focuses on selectively assigning RSUs to a subset of intersections while leaving others without RSU coverage.

In [12], the authors analyze the safety message broadcast performance of the IEEE 802.11p standard in urban road intersection environments, considering different communication and carrier sensing ranges, and divides the intersection area using partitioning methods. The study finds that when broadcasting vehicles are far from the center of the intersection, the overall transmission rate is very low. To improve this situation, the article proposes a scheme to relay safety messages at the intersection center using RSUs, and demonstrates performance enhancements using omnidirectional antennas and bidirectional sector antennas. The results indicate that relaying with omnidirectional antennas can moderately improve the overall transmission rate, while using sector antennas can achieve significant performance improvements.

Furthermore, Nidhi and Lobiyal [13] evaluate the performance of vehicle-to-infrastructure communication at intersections in dense urban areas with deployed RSUs, providing insights into communication efficiency under such conditions. The research assesses vehicle-to-infrastructure communication by studying vehicle mobility and communication patterns, using indicators like packet delivery ratio, packet loss, routing overhead, and end-to-end delay. The results show that higher vehicle density leads to decreased packet delivery ratio and increased packet loss, indicating worsened congestion and packet collisions. Routing overhead varies inconsistently with vehicle count, and end-to-end delay rises with traffic. These findings highlight challenges in RSU deployment in dense areas, guiding future research on efficient RSU strategies and suitable vehicle-to-infrastructure models for different density locations.

### 2.3. RSU Deployment for Single Highways

RSU deployment along single highways constitutes another active area of research interest. In [14], the authors propose a strategy called capacity maximization placement (CMP), which not only considers two modes of vehicle access to RSUs, direct access and multi-hop relay access, but also takes into account factors such as wireless interference, non-uniform distribution of vehicles, and vehicle speeds. By establishing an integer linear programming model, the CMP strategy can adapt to different vehicle distributions and speeds, optimizing the deployment locations and quantities of RSUs. Compared to simple uniform distribution deployment and hotspot area deployment, the CMP strategy achieves better performance under different vehicle distribution and speed conditions. Especially in complex road environments with uneven vehicle distribution and multiple lanes, exits, or intersections, the CMP strategy can flexibly adjust RSU deployment according to actual conditions to achieve the optimal balance between cost-effectiveness and network performance. However, the model used in the article may oversimplify the complexity of the real world. For example, the mobility and distribution of vehicles may be influenced by various factors that may not be fully reflected in the model.

In [15], the authors investigate how to minimize transmission delays and ensure good network capacity in VANETs in highway-like scenarios by optimizing the layout of RSUs. Building upon the CMP algorithm in [14], the author abandons the throughput maximization objective used in the CMP algorithm and instead formulates a finalization equation based on the transmission delay between vehicles and RSUs to establish an RSU deployment scheme. This scheme considers both direct transmission and multi-hop relaying between vehicles and RSUs, and employs VanetMobiSim to generate traffic mobility patterns, followed by simulation validation. However, similar to [14], this study may suffer from the same shortcomings: oversimplification of real-world complexity in the models used, potential influences on vehicle mobility and distribution by various factors not fully represented in the model.

Aslam and Zou [16] delve into optimizing the layout of RSUs along highways in VANETs to minimize the average time for vehicles to report events to nearby RSUs. The author proposes a dynamic process called the “balloon optimization method”, which finds the optimal solution by simulating the natural expansion of multiple balloons in two-dimensional space, with each balloon representing the coverage area of an RSU. Through preliminary evaluation, the balloon optimization method demonstrates optimal or near-optimal performance compared to exhaustive methods and can be applied to optimize RSU deployment on highways. However, the theoretical derivation provided in this article is incomplete, and it only investigates the RSU deployment problem on a single road segment.

Meanwhile, in [17], the authors present a cost-effective RSU deployment model based on real data, aiming to enhance connectivity in VANETs within highway scenarios. By utilizing graph theory concepts and real-data-based random vehicle positioning in simulation environments, the model can determine the minimum number of RSU deployment locations to maintain connectivity on any given segment of the highway. Research results demonstrate that when real data are available, this model can effectively provide RSU deployment for any segment of the highway, ensuring coverage for all vehicles. However, the model in this article may be based on ideal assumptions regarding the randomness and uniformity of vehicle distribution, potentially deviating from the realities of the real world.

Ge and Chung [18] employ an approximation algorithm to evenly distribute RSUs along highway segments, aiming to enhance VANET connectivity. These authors investigate the efficient deployment of RSUs in intelligent highway environments with the aim of providing excellent connectivity in VANETs. They propose an approximately optimal RSU deployment scheme designed to estimate the minimum number of RSUs required to ensure VANET connectivity within a given threshold, taking into account equidistant deployment of RSUs. The performance of the proposed scheme is evaluated by applying approximation algorithms to allocate RSU locations and computing the connectivity probability of VANETs. Simulation results indicate that for each vehicle network with *N* vehicles, there exists a threshold *M* corresponding to the number of deployed RSUs, and as the number of RSUs increases, the growth in the connectivity probability becomes slower.

Finally, in [19], a vehicle clustering algorithm is proposed to determine RSU locations along highway segments. The study analyzes the impact of RSU-to-vehicle communication radius, mixed traffic density, and different connected and autonomous vehicle penetration rates on the spacing of RSU deployments. However, this work shares the same weakness as the aforementioned highway RSU deployment schemes—only single highways are considered in the problem.

Based on the review of related work above, it is clear that most research on RSU deployment has concentrated on urban environments, urban intersections, and single highways. These studies typically analyze traffic densities statically, which may not accurately represent real-world scenarios. Due to substantial differences in layout and traffic characteristics among highway intersections, single highways, and urban intersections, there is a lack of specific modeling and theoretical derivation tailored for highway intersections. As a result, RSU deployment solutions proposed in the existing literature cannot be directly applied to deploy RSUs for highway intersections. Therefore, there is an urgent need to propose an RSU deployment scheme specifically designed for highway intersections. Given that event reporting delay is a critical performance measure influencing RSU deployment, which, in turn, enhances traffic safety and supports autonomous driving services, we have developed a delay-oriented RSU deployment approach for highway intersections.

## 3. System Model

**Figure 1:**
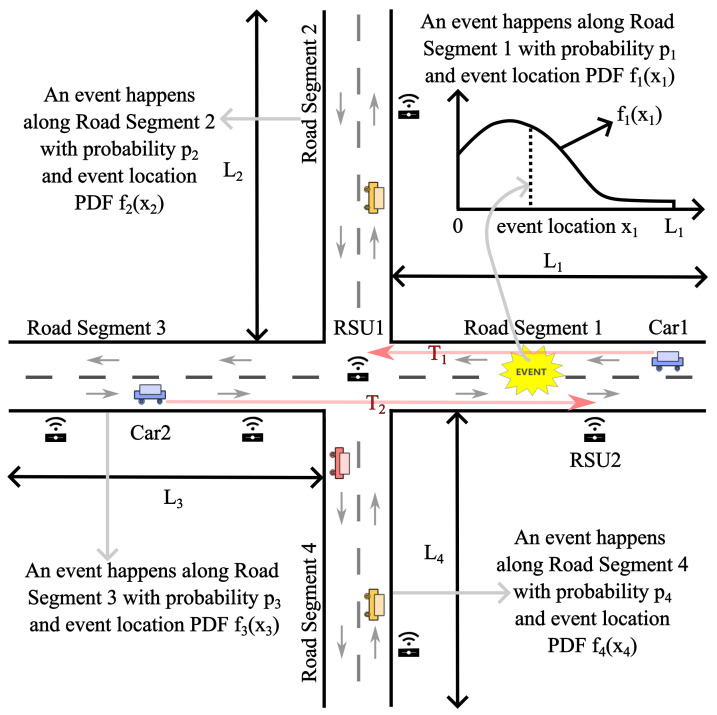
A schematic diagram of a highway intersection, and an illustrative example of the event reporting delays of single directions (experienced separately by RSU1 and RSU2 since the event occurs), $T_{1}$ and $T_{2}$.

In this paper, we consider an intersection connecting four road segments, as in Figure 1, denoted as RS1, RS2, RS3, and RS4, respectively. Let $L_{1}$, $L_{2}$, $L_{3}$, and $L_{4}$ represent the lengths of these road segments, and *N* denote the total number of RSUs to be deployed along them. We assume the traffic flows in each direction of a road segment follow Poisson point processes with rates $\lambda_{1}$, $\lambda_{2}$, $\lambda_{3}$, and $\lambda_{4}$ [9,20,21,22]. Vehicle speeds on the road segments, denoted as $v_{1}\left(x_{1}\right)$, $v_{2}\left(x_{2}\right)$, $v_{3}\left(x_{3}\right)$, and $v_{4}\left(x_{4}\right)$, where $x_{1}$, $x_{2}$, $x_{3}$, and $x_{4}$ denote the coordinates along the respective road segments, with the intersection as the origin, are assumed to be constant and equal to $v_{1}$, $v_{2}$, $v_{3}$, and $v_{4}$ for their respective segments. An event may occur along one of the four road segments of an intersection, with respective probabilities denoted as $p_{1}$, $p_{2}$, $p_{3}$, and $p_{4}$, where $p_{1}+p_{2}+p_{3}+p_{4}=1$. The PDFs describing event locations along RS1, RS2, RS3, and RS4 are represented by $f_{1}\left(x_{1}\right)$, $f_{2}\left(x_{2}\right)$, $f_{3}\left(x_{3}\right)$, and $f_{4}\left(x_{4}\right)$, respectively. The event location PDF for the highway intersection is formulated as

(1) $$f\left(x_{1},x_{2},x_{3},x_{4}\right)=\left\{\begin{array}{cc} p_{1}f_{1}\left(x_{1}\right), & \mathrm{if}\;x_{1}\in \left[0,L_{1}\right],x_{2}=x_{3}=x_{4}=0,\\ p_{2}f_{2}\left(x_{2}\right), & \mathrm{if}\;x_{2}\in \left(0,L_{2}\right],x_{1}=x_{3}=x_{4}=0,\\ p_{3}f_{3}\left(x_{3}\right), & \mathrm{if}\;x_{3}\in \left(0,L_{3}\right],x_{1}=x_{2}=x_{4}=0,\\ p_{4}f_{4}\left(x_{4}\right), & \mathrm{if}\;x_{4}\in \left(0,L_{4}\right],x_{1}=x_{2}=x_{3}=0,\\ 0, & \mathrm{otherwise}. \end{array}\right.$$

This probabilistic model integrates the PDFs of event locations along the road segments within a highway intersection, providing a comprehensive description of the spatial distribution of events in the surrounding area.

In our derivation, we consider the worst-case scenario of event reporting delay, where the traffic density is so sparse that relaying transmissions by other vehicles is not feasible. We opt for this extreme case because highway systems typically have few and scattered vehicles, and this choice ensures that the RSUs are deployed to meet the quality-of-service requirements of VANETs even under the most challenging communication conditions.

A key precondition of our scheme is that one RSU is placed at the intersection, with the remaining RSU locations determined through deployment calculations. We adopt this premise because the RSU positioned at the intersection can communicate with vehicles on all four road segments, enhancing its utility. Moreover, in cases where RSUs along the road segments become disconnected due to malfunctions, the intersection RSU can serve as a backup connection for all four road segments. This redundancy aligns with the quick recovery communication requirement of safety applications.

## 4. RSU Deployment Scheme

Our RSU deployment scheme unfolds in two steps. First, we calculate the optimal RSU locations along one road segment given that *n* RSUs are to be positioned along it, excluding the intersection RSU. Next, we determine the number of RSUs assigned to each of the four road segments by minimizing the overall expected event reporting delay at the intersection. This is expressed as $n_{1}+n_{2}+n_{3}+n_{4}+1=N$, where $n_{1}$, $n_{2}$, $n_{3}$, and $n_{4}$ represent the numbers of RSUs assigned to RS1, RS2, RS3, and RS4, respectively.

### 4.1. Optimal Locations for RSUs Deployed on One Road Segment

For a highway road segment with length *L*, connected to an intersection, where *n* RSUs are to be deployed (aside from the one placed at the intersection), the locations of the RSUs are determined by minimizing the expected event reporting delay, denoted by $E[T_{RS}|n]$. Here, the event reporting delay $T_{RS}$ is a random variable, and its expected value depends on the RSU locations along the road segment, denoted by $r_{0},r_{1},\cdots ,r_{n}$.

**Figure 2:**
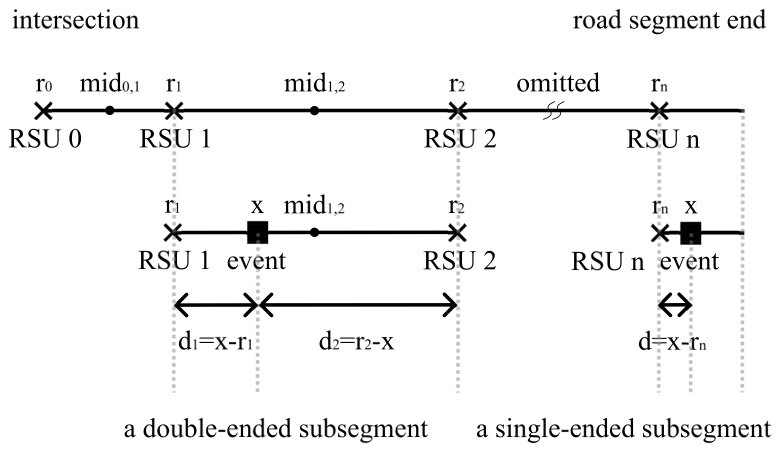
An illustration of n+1 RSUs deployed along a road segment, including the one positioned at the intersection.

In the provided figure (Figure 2), there are n+1 RSUs deployed along the road segment, including the RSU located at the intersection. The road segment is divided into subsegments by these RSUs, resulting in two types: double-ended subsegments and single-ended subsegments. Double-ended subsegments have RSUs on both ends, while single-ended subsegments are located at the end of the road segment and have only one RSU. In double-ended subsegments, the middle points are denoted by $mid_{0,1},mid_{1,2},\cdots ,mid_{n-1,n}$, where $mid_{i,i+1}=\left(r_{i}+r_{i+1}\right)/2$, with i∈{0,1,⋯,n−1}. Events occurring along these subsegments, depicted as black squares in Figure 2, are reported to an RSU at either end (or the only end) of the subsegment. The event location is represented by *x*, and its PDF is denoted by f(x). Both directions’ traffic flows follow Poisson point processes with rates λ, and vehicles travel at a constant speed *v*.

#### 4.1.1. Event Reporting Delay on Double-Ended Subsegments

In the example of the double-ended subsegment shown in Figure 2 (the subsegment between RSU1 and RSU2), an event occurs at position *x*, where $x\in [r_{1},r_{2})$. Its distance to the RSU on the left is denoted by $d_{1}$, where $d_{1}=x-r_{1}$, and its distance to the RSU on the right is denoted by $d_{2}$, where $d_{2}=r_{2}-x$. Let $T_{1}$ and $T_{2}$ denote the random variables representing the event reporting delays experienced separately by RSU1 and RSU2 since the event occurs (an illustrative example of $T_{1}$ and $T_{2}$ can also be found in Figure 1, in which Car1 report the event to RSU1 and Car2 report the event to RSU2, following the corresponding red arrows in Figure 1), and 

(2) $$T_{1}=Y_{1}+\frac{d_{1}}{v}$$

(3) $$T_{2}=Y_{2}+\frac{d_{2}}{v}$$

The random variables $Y_{1}$ and $Y_{2}$ represent the time it takes for the first passing vehicles, heading towards RSU1 and RSU2, respectively, to reach the event location after its occurrence. These variables follow an exponential distribution with the rate parameter λ. Additionally, $\frac{d_{1}}{v}$ and $\frac{d_{2}}{v}$ represent the time periods required for the vehicles to transmit the event messages to RSU1 and RSU2, respectively, where *v* is the constant speed of the vehicles. The conditional PDFs, denoted as $f_{T_{1}}\left(t|x\right)$ and $f_{T_{2}}\left(t|x\right)$, and the conditional cumulative distribution functions (CDFs), denoted as $F_{T_{1}}\left(t|x\right)$ and $F_{T_{2}}\left(t|x\right)$, of $T_{1}$ and $T_{2}$ are given as follows:

For $T_{1}$, the event reporting delay experienced by RSU1, the conditional PDF $f_{T_{1}}\left(t|x\right)$ is determined by the time it takes for the first vehicle (driving from right to left) to reach the event after the event occurs, plus the time it takes for the vehicle to process and transmit the event message to RSU1. This can be expressed as

(4) $$f_{T_{1}}\left(t|x\right)=\lambda e^{-\lambda (t-\frac{d_{1}}{v})},\;\frac{d_{1}}{v}\leq t$$

The conditional CDF $F_{T_{1}}\left(t|x\right)$ can be obtained by integrating the PDF

(5) $$F_{T_{1}}\left(t|x\right)=1-e^{-\lambda (t-\frac{d_{1}}{v})},\;\frac{d_{1}}{v}\leq t$$

Similarly, for $T_{2}$, the event reporting delay experienced by RSU2, the conditional PDF $f_{T_{2}}\left(t|x\right)$ is given by

(6) $$f_{T_{2}}\left(t|x\right)=\lambda e^{-\lambda (t-\frac{d_{2}}{v})},\;\frac{d_{2}}{v}\leq t$$

And the conditional CDF $F_{T_{2}}\left(t|x\right)$ is

(7) $$F_{T_{2}}\left(t|x\right)=1-e^{-\lambda (t-\frac{d_{2}}{v})},\;\frac{d_{2}}{v}\leq t.$$

Hence, the event reporting delay $T_{DS}=\min\left\{T_{1},T_{2}\right\}$, where the subscript ‘DS’ represents ‘double-ended subsegment’, and its conditional PDF, $f_{T_{DS}}\left(t|x\right)$, has two cases.

Case 1: when $x<mid_{1,2}$, i.e., $d_{1}<d_{2}$,

(8) $$\begin{array}{cc} \; & f_{T_{{DS}_{left}}}\left(t|x\right)\\ \; & =\left\{\begin{array}{cc} f_{T_{1}}\left(t|x\right)\left(1-F_{T_{2}}\left(t|x\right)\right)\\ \;+f_{T_{2}}\left(t|x\right)\left(1-F_{T_{1}}\left(t|x\right)\right), & \frac{d_{2}}{v}<t\\ f_{T_{1}}\left(t|x\right), & \frac{d_{1}}{v}\leq t\leq \frac{d_{2}}{v}\\ 0, & \;\;t<\frac{d_{1}}{v} \end{array}\right.\\ \; & =\left\{\begin{array}{cc} 2\lambda e^{-\lambda (2t-\frac{d_{1}+d_{2}}{v})}, & \;\;\;\;\frac{d_{2}}{v}<t\\ \lambda e^{-\lambda (t-\frac{d_{1}}{v})}, & \;\;\;\;\frac{d_{1}}{v}\leq t\leq \frac{d_{2}}{v}\\ 0, & \;\;\;\;\;\;t<\frac{d_{1}}{v}. \end{array}\right. \end{array}$$

Therefore, the conditional expected value of the event reporting delay on the left half of this subsegment, $E[T_{{DS}_{left}}|x]$, under Case 1 is

(9) $$\begin{array}{cc} \; & E[T_{{DS}_{left}}|x]\\ & =\int_{\frac{d_{1}}{v}}^{\frac{d_{2}}{v}}\lambda te^{-\lambda (t-\frac{d_{1}}{v})}dt+\int_{\frac{d_{2}}{v}}^{+\infty }2\lambda te^{-\lambda (2t-\frac{d_{1}+d_{2}}{v})}dt\\ \; & =\frac{d_{1}}{v}+\frac{1}{\lambda }-\frac{1}{2\lambda }e^{-\lambda \frac{d_{2}-d_{1}}{v}}. \end{array}$$

Case 2: when $x>mid_{1,2}$, i.e., $d_{1}>d_{2}$,

(10) $$\begin{array}{cc} \; & f_{T_{{DS}_{right}}}\left(t|x\right)\\ \; & =\left\{\begin{array}{cc} f_{T_{1}}\left(t|x\right)\left(1-F_{T_{2}}\left(t|x\right)\right)\\ \;+f_{T_{2}}\left(t|x\right)\left(1-F_{T_{1}}\left(t|x\right)\right), & \frac{d_{1}}{v}<t\\ f_{T_{2}}\left(t|x\right), & \frac{d_{2}}{v}\leq t\leq \frac{d_{1}}{v}\\ 0, & \;\;t<\frac{d_{2}}{v} \end{array}\right.\\ \; & =\left\{\begin{array}{cc} 2\lambda e^{-\lambda (2t-\frac{d_{1}+d_{2}}{v})}, & \;\;\;\;\frac{d_{1}}{v}<t\\ \lambda e^{-\lambda (t-\frac{d_{2}}{v})}, & \;\;\;\;\frac{d_{2}}{v}\leq t\leq \frac{d_{1}}{v}\\ 0, & \;\;\;\;\;\;t<\frac{d_{2}}{v}. \end{array}\right. \end{array}$$

Therefore, the conditional expected value of the event reporting delay on the right half of this subsegment, denoted by $E[T_{{DS}_{right}}|x]$, under Case 2 is

(11) $$\begin{array}{cc} \; & E[T_{{DS}_{right}}|x]\\ & =\int_{\frac{d_{2}}{v}}^{\frac{d_{1}}{v}}\lambda te^{-\lambda (t-\frac{d_{2}}{v})}dt+\int_{\frac{d_{1}}{v}}^{+\infty }2\lambda te^{-\lambda (2t-\frac{d_{1}+d_{2}}{v})}dt\\ \; & =\frac{d_{2}}{v}+\frac{1}{\lambda }-\frac{1}{2\lambda }e^{-\lambda \frac{d_{1}-d_{2}}{v}}. \end{array}$$

The same methodology can be applied to assess event reporting delays in other double-ended subsegments as well.

#### 4.1.2. Event Reporting Delay on Single-Ended Subsegments

Similarly, in the single-ended subsegment example illustrated in Figure 2 (the subsegment at the end of the road segment with only RSU*n* at the left end), an event occurs at position *x*, where $x\in [r_{n},L]$. Its distance to the RSU on the left is denoted by *d*, where $d=x-r_{n}$. The conditional probability density function (PDF) of the event reporting delay on this subsegment, denoted as $f_{T_{SS}}\left(t|x\right)$, is given by

(12) $$f_{T_{SS}}\left(t|x\right)=\lambda e^{-\lambda (t-\frac{d}{v})}$$

where the subscript ‘SS’ represents ‘single-ended subsegment’. Therefore, the conditional expected value of the event reporting delay on this subsegment, denoted by $E[T_{SS}|x]$, is

(13) $$E\left[T_{SS}|x\right]=\int_{\frac{d}{v}}^{+\infty }\lambda te^{-\lambda (t-\frac{d}{v})}dt=\frac{d}{v}+\frac{1}{\lambda }.$$

The expected value of the event reporting delay along the entire road segment, considering the deployment of *n* RSUs and the event location PDF, can be expressed as $E[T_{RS}|n]$

(14) $$\begin{array}{cc} E[T_{RS}|n]= & \sum_{i=0}^{n-1}\int_{r_{i}}^{mid_{i,i+1}}E\left[T_{{DS}_{left}}|x\right]f\left(x\right)dx\\ & \;+\sum_{i=0}^{n-1}\int_{mid_{i,i+1}}^{r_{i+1}}E\left[T_{{DS}_{right}}|x\right]f\left(x\right)dx\\ & \;\;+\int_{r_{n}}^{L}E\left[T_{SS}|x\right]f\left(x\right)dx \end{array}$$

and the optimal locations for the *n* RSUs are found by minimizing $E[T_{RS}|n]$

(15) $$\begin{array}{ccc} \underset{r_{1},r_{2},\cdots ,r_{n}}{\arg\min}\; & E[T_{RS}|n] & \\ s.t.\;\; & r_{1},r_{2},\cdots ,r_{n}\in \left(0,L\right) & \\ \; & r_{1}<r_{2}<\cdots <r_{n}. \end{array}$$

### 4.2. Numbers of RSUs Assigned to the Four Road Segments

Given that $n_{1}$, $n_{2}$, $n_{3}$, and $n_{4}$ RSUs are assigned to RS1, RS2, RS3, and RS4, respectively, and the RSUs are deployed at their optimal locations along each road segment calculated from Equation (15), the expected event reporting delay for the entire intersection can be determined as

(16) $$\begin{array}{cc} E[T_{int}]= & p_{1}E\left[T_{RS1}|n_{1}\right]+p_{2}E\left[T_{RS2}|n_{2}\right]\\ & \;\;+p_{3}E\left[T_{RS3}|n_{3}\right]+p_{4}E\left[T_{RS4}|n_{4}\right] \end{array}$$

where the subscript ‘int’ represents the word ‘intersection’. Therefore, the optimal numbers of RSUs to distribute to the four road segments are determined by minimizing the expected event reporting delay for the entire intersection

(17) $$\begin{array}{ccc} \underset{n_{1},n_{2},n_{3},n_{4}}{\arg\min}\; & E[T_{int}] & \\ s.t.\;\; & n_{1},n_{2},n_{3},n_{4}\in N & \\ \; & n_{1}+n_{2}+n_{3}+n_{4}+1=N. \end{array}$$

## 5. RSU Deployment Algorithm

The cooperative walking algorithm, outlined in Algorithm 1, is developed to implement our scheme. Two stages are designed: Stage 1: This stage aims at finding optimal RSU locations with the minimum expected event reporting delay $E[T_{RS}|n]$ for each road segment connected to the intersection, considering different values of *n* (excluding the RSU at the intersection). Stage 2: In this stage, the algorithm finds the optimal combination of RSU numbers for the four road segments to achieve the minimum expected event reporting delay for the entire intersection.
**Algorithm 1** The Cooperative Walking Algorithm for Highway Intersection RSU Deployment**STAGE 1:** RSU Deployment for Each Road Segment**(1)** n=0 (only one RSU at the intersection)    Calculate $E[T_{RS}|0]$**(2)** n=1, start from $r_{1}=0$    $r_{1}$ increments, until $\min E[T_{RS}|1]$ is reached**(3)** n=m>1, start from $r_{1}=0$ and $r_{2},\cdots ,r_{m}$ equal to the optimal locations of $r_{1},\cdots ,r_{m-1}$ in the n=m−1 case    $r_{1}$ increments, until $E[T_{RS}|m]$ stops decreasing or $r_{1}=r_{2}$    **do**       **for i=m:−1:1**           $r_{i}$ increments, until $E[T_{RS}|m]$ stops decreasing or $r_{i}=r_{i+1}$ ($r_{i}=L$, when i=m)    **while** (there is any RSU location change)    Calculate $\min E[T_{RS}|m]$ **STAGE 2:** RSU Deployment for the Intersection**(4)** Find all combinations of $n_{1}+n_{2}+n_{3}+n_{4}+1=N$**(5)** Calculate $E[T_{int}]$ in Equation (16) for all combinations**(6)** The combination with the smallest $E[T_{int}]$ is chosen**(7)** The RSUs are deployed along the four road segments according to the combination of RSU numbers and the corresponding optimal locations found in Stage 1

In Stage 1, when n=0, there are no RSUs to deploy, and events are reported to the RSU at the intersection; the expected event reporting delay is $E[T_{RS}|0]$. When n=1, there is one RSU to deploy, in addition to the RSU at $r_{0}$. The initial location of this RSU is $r_{1}=0$, and it increments until reaching the minimum $E[T_{RS}|1]$. When n=m>1, there are *m* RSUs to locate, apart from the RSU at $r_{0}$. The initial locations of these *m* RSUs are as follows: $r_{1}=0$, and $r_{2},\cdots ,r_{m}$ are set equal to the optimal locations of $r_{1},\cdots ,r_{m-1}$ in the n=m−1 case. Once the initial locations are set, $r_{1}$ increments while the other RSU locations remain constant until $E[T_{RS}|m]$ ceases to decrease or $r_{1}=r_{2}$. It is important to note that RSUs are not allowed to reverse their orders during this process.

Following this, a do-while loop initiates; within one loop iteration, for *i* decreasing from *m* to 1, the procedure of incrementing $r_{i}$ is repeated until $E[T_{RS}|m]$ no longer decreases or $r_{i}=r_{i+1}$ ($r_{i}=L$ when i=m). This do-while loop persists as long as there is any change in RSU location, indicating a decrease in the expected event reporting delay in the preceding loop. Upon exiting the loop, the minimum $E[T_{RS}|m]$ is calculated from $r_{1},r_{2},\cdots ,r_{m}$, and these locations constitute the optimal RSU positions along this road segment for n=m.

In Stage 2, all combinations of $n_{1}+n_{2}+n_{3}+n_{4}+1=N$ are enumerated, and the expected intersection event reporting delays, $E[T_{int}]$, are computed for each combination. The combination yielding the smallest $E[T_{int}]$ is selected. RSUs are then deployed along the four road segments based on the RSU numbers in the chosen combination and the corresponding optimal locations determined in Stage 1.

The most innovative aspect of our algorithm lies in Stage 1 Step (3), which also inspired the name “Cooperative Walking” for the algorithm. In this step, when n=m>1, m−1 of the RSU locations are initialized based on the optimal locations found in the n=m−1 scenario. To deploy one additional RSU along the road segment, occupying the location of $r_{1}$, all other m−1 RSUs’ coordinates must increment to minimize $E[T_{RS}|m]$. Consequently, the coordinates of the *m* RSUs take turns incrementing in a looping fashion, as long as $E[T_{RS}|m]$ decreases and the RSU order remains unchanged. This cooperative process resembles RSUs taking turns to walk into their optimal positions, thus inspiring the name “Cooperative Walking” for the algorithm.

## 6. Example Results

In this section, we provide two examples to illustrate the effectiveness of our RSU deployment approach. We juxtapose the delay performance of our scheme with two alternative strategies: one where RSUs are uniformly distributed and another based on the balloon method proposed in [16].

### 6.1. Events Occur with Truncated Normal Distributions along the Road Segments

**Figure 3:**
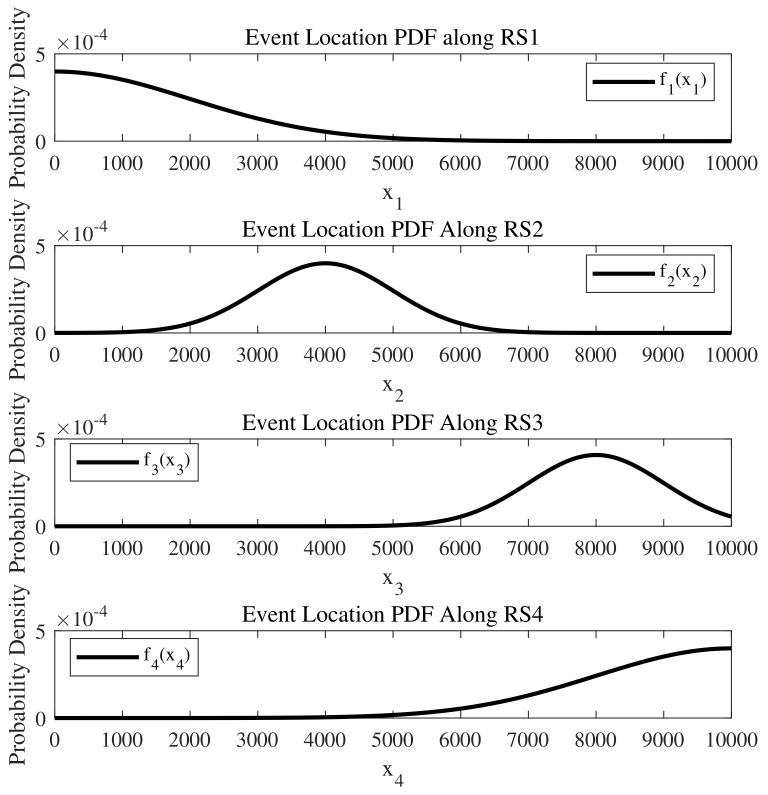
The truncated normal distributions along the four road segments represent the probability distribution of event occurrences within an intersection where events may take place.

In this example, we assume that the PDFs of event locations along the four road segments follow truncated normal distributions. The PDF of a truncated normal distribution defined on the interval [a,b] is given by

(18) $$f\left(x\right)=\left\{\begin{array}{cc} \frac{\frac{1}{\sigma \sqrt{2\pi }}e^{-\frac{1}{2}{\left(\frac{x-\mu }{\sigma }\right)}^{2}}}{\int_{a}^{b}\frac{1}{\sigma \sqrt{2\pi }}e^{-\frac{1}{2}{\left(\frac{x-\mu }{\sigma }\right)}^{2}}dx}, & \;x\in [a,b]\\ 0, & \;otherwise \end{array}\right.$$

where μ and σ represent the mean and standard deviation of the normal distribution, respectively. Let $\mu_{1}=0$, $\sigma_{1}=2000$, $\mu_{2}=4000$, $\sigma_{2}=1000$, $\mu_{3}=8000$, $\sigma_{3}=1000$, $\mu_{4}=10,000$, $\sigma_{4}=2000$, all measured in meters, and the distributions are defined on the interval [0,10,000] m, as depicted in Figure 3. Additionally, let $L_{1}=L_{2}=L_{3}=L_{4}=10,000$ m, $\lambda_{1}=\lambda_{2}=\lambda_{3}=\lambda_{4}=0.05$, $v_{1}=v_{2}=v_{3}=v_{4}=30$ m/s, $p_{1}=0.2$, $p_{2}=0.3$, $p_{3}=0.4$, and $p_{4}=0.1$.

**Figure 4:**
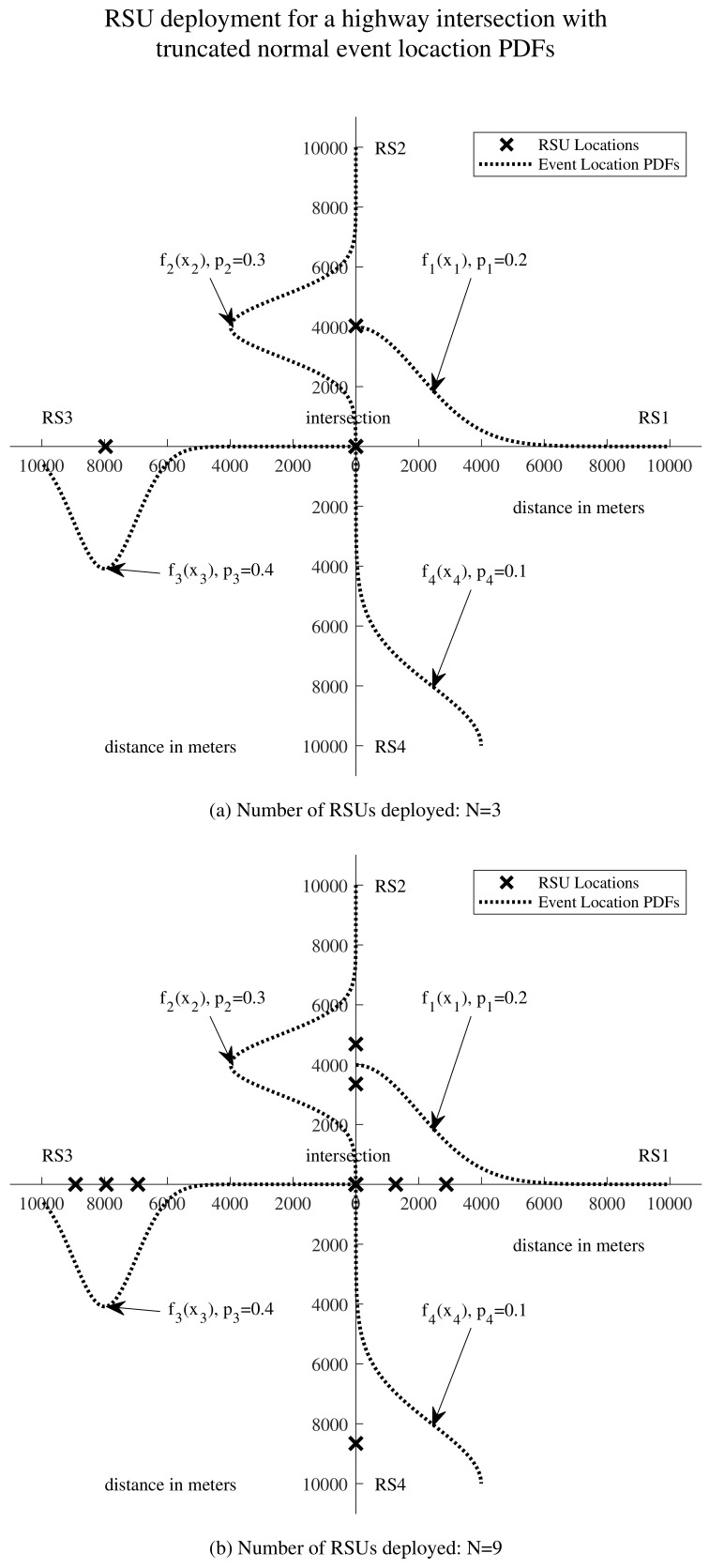
Illustrative example of delay-oriented RSU deployment for highway intersections. Events occur with truncated normal distributions along the four road segments. (**a**) Deployment with 3 RSUs at the intersection. (**b**) Deployment with 9 RSUs at the intersection.

Figure 4 illustrates delay-oriented RSU deployment at highway intersections, depicting a simplified layout of a highway intersection with four road segments and varying numbers of deployed RSUs. In Figure 4a, the scenario with N=3 is depicted, while in Figure 4b, the scenario with N=9 is presented. The black crosses denote the optimal locations for the RSUs. Additionally, the truncated normal event location probability density functions (PDFs) are plotted above their corresponding road segments. Under our RSU deployment scheme, the RSUs are positioned more densely in areas with higher event probabilities. For instance, in Figure 4b, on RS3, the PDF $f_{3}\left(x_{3}\right)$ exhibits a single mode at 8000, with $p_{3}=0.4$, the highest among $p_{1}$, $p_{2}$, $p_{3}$, and $p_{4}$. This indicates a higher likelihood of events occurring around 8000 m on RS3 compared to other areas with lower event probabilities. Consequently, three out of nine RSUs are deployed at 6942, 7948, and 8926 m along RS3.

**Figure 5:**
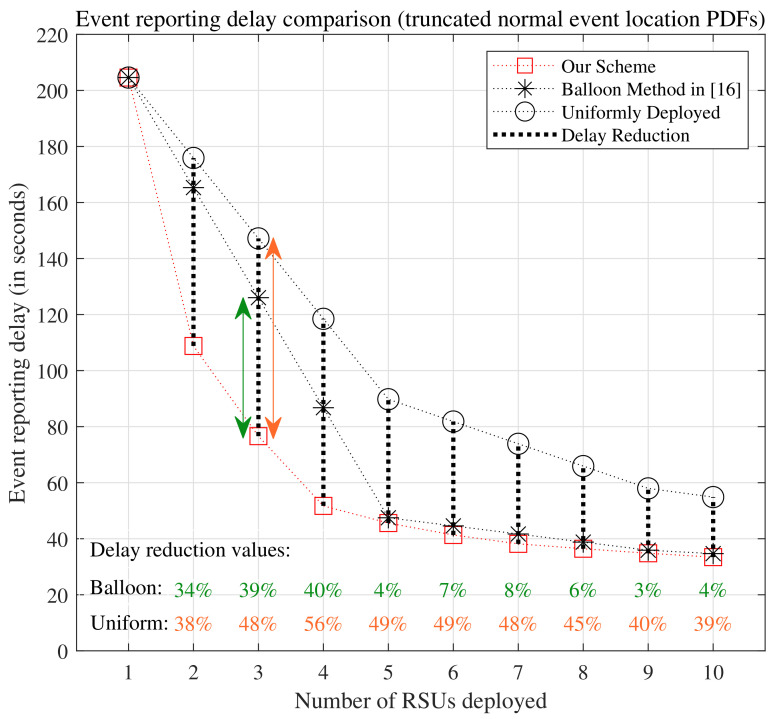
Comparison of event reporting delays of the uniformly distributed scheme, the balloon method in [16], and our scheme, when conducted under the scenario where events occur with truncated normal distributions along the road segments.

Figure 5 illustrates the comparison of event reporting delays of the uniformly distributed scheme, the balloon method in [16], and our scheme for different values of *N* ranging from 1 to 10. The delay reduction is calculated as the ratio of the delay difference of the compared scheme (the uniformly distributed scheme or the balloon method in [16]) and our scheme over the delay in the compared scheme. Detailed delay values can be found in Table 1. Notably, the delay reduction values range from 38% to 56% when compared with the event reporting delays of the uniformly distributed scheme, and from 3% to 40% when compared with the balloon method, for N>1, indicating significant improvements. Our scheme demonstrates notable performance enhancements in event reporting delay. Notice in Figure 5 that for N≥5, the reduction in delay achieved by our scheme compared to the balloon method is not as pronounced as for N≤4. This is because when a sufficient number of RSUs are deployed in the vicinity where events are likely to occur, a larger proportion of the event reporting delays are primarily determined by the time it takes for the first vehicle to reach the event after its occurrence. The processing and transmission time for the event message to the RSU constitutes a smaller portion of the overall delay. As vehicle arrival rates increase, the delay reduction values for N≥5 would also increase.

### 6.2. Events Occur with Uniform Distributions along the Road Segments

In this second example, the PDFs of event locations along the four road segments are assumed to follow uniform distributions. The PDF of a uniform distribution defined on the interval [a,b] is given by

(19) $$f\left(x\right)=\left\{\begin{array}{cc} \frac{1}{b-a}, & \;x\in [a,b]\\ 0, & \;otherwise. \end{array}\right.$$

**Figure 6:**
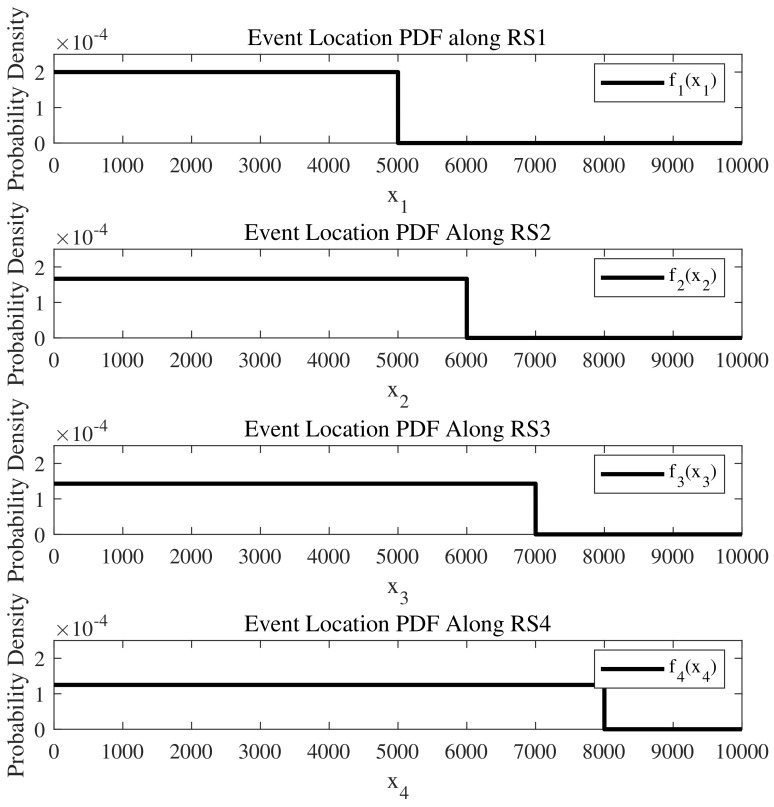
The uniform distributions along the four road segments represent the probability distribution of event occurrences within an intersection where events may take place.

We set $a_{1}=a_{2}=a_{3}=a_{4}=0$, $b_{1}=5000$, $b_{2}=6000$, $b_{3}=7000$, $b_{4}=8000$, all in meters, as depicted in Figure 6. Similar to the previous example, $L_{1}=L_{2}=L_{3}=L_{4}=10,000$ m, $\lambda_{1}=\lambda_{2}=\lambda_{3}=\lambda_{4}=0.05$, $v_{1}=v_{2}=v_{3}=v_{4}=30$ m/s, $p_{1}=0.2$, $p_{2}=0.3$, $p_{3}=0.4$, and $p_{4}=0.1$.

**Figure 7:**
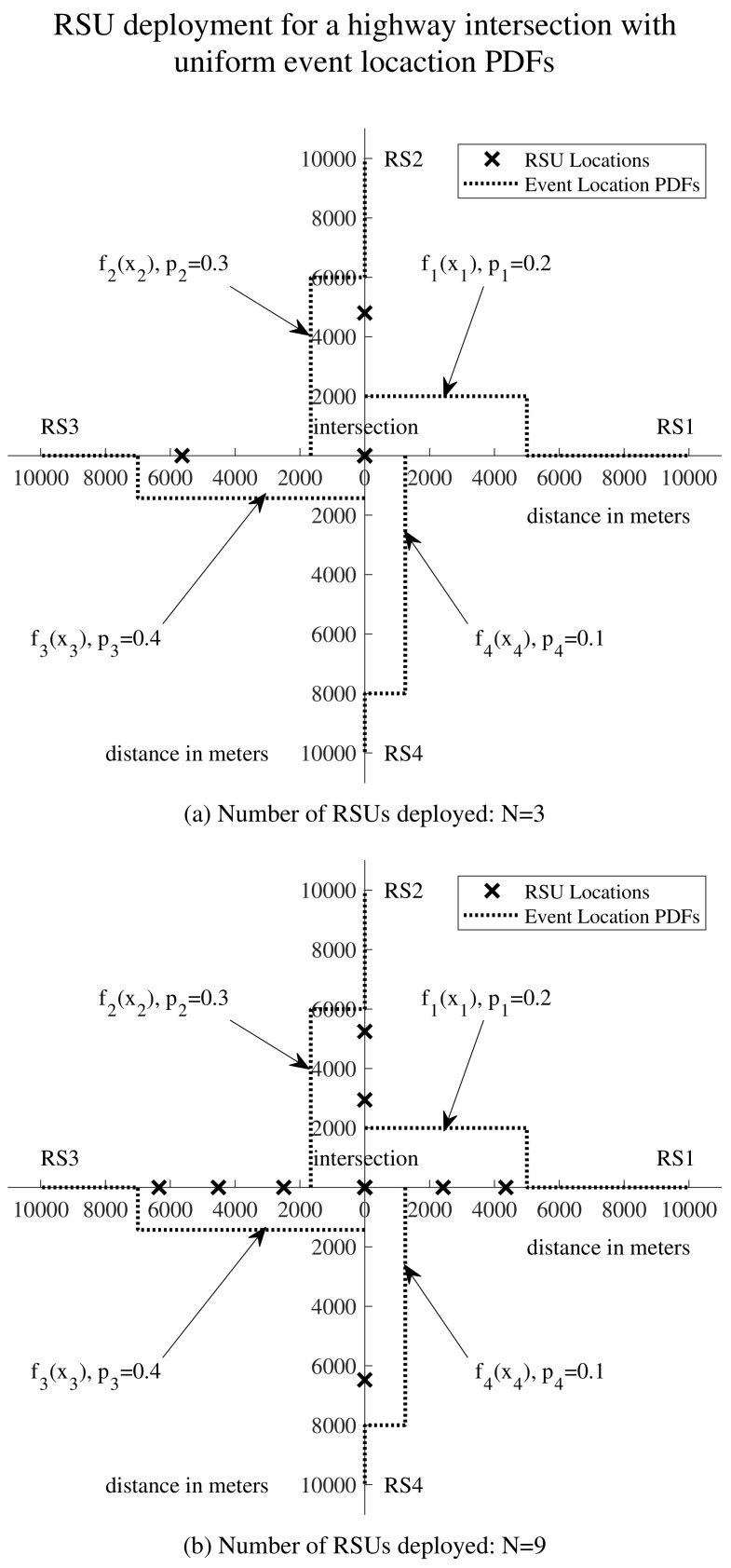
Illustrative example of delay-oriented RSU deployment for highway intersections. Events occur with uniform distributions along the four road segments. (**a**) Deployment with 3 RSUs at the intersection. (**b**) Deployment with 9 RSUs at the intersection.

Figure 7 illustrates delay-oriented RSU deployment at highway intersections, depicting an intersection with four road segments featuring varying numbers of deployed RSUs. In Figure 7a, the scenario with N=3 is depicted, while in Figure 7b, the scenario with N=9 is presented. The black crosses represent the optimal locations for the RSUs. Additionally, the uniform event location probability density functions (PDFs) are plotted above their corresponding road segments. Under our RSU deployment scheme, the RSUs are positioned more densely in areas with higher event probabilities. For example, in Figure 7b, on RS3, $f_{3}\left(x_{3}\right)$ is defined on [0,7000], and $p_{3}=0.4$, the highest among $p_{1}$, $p_{2}$, $p_{3}$, and $p_{4}$. Consequently, three out of nine RSUs are deployed at 2499, 4516, and 6349 m along RS3.

**Figure 8:**
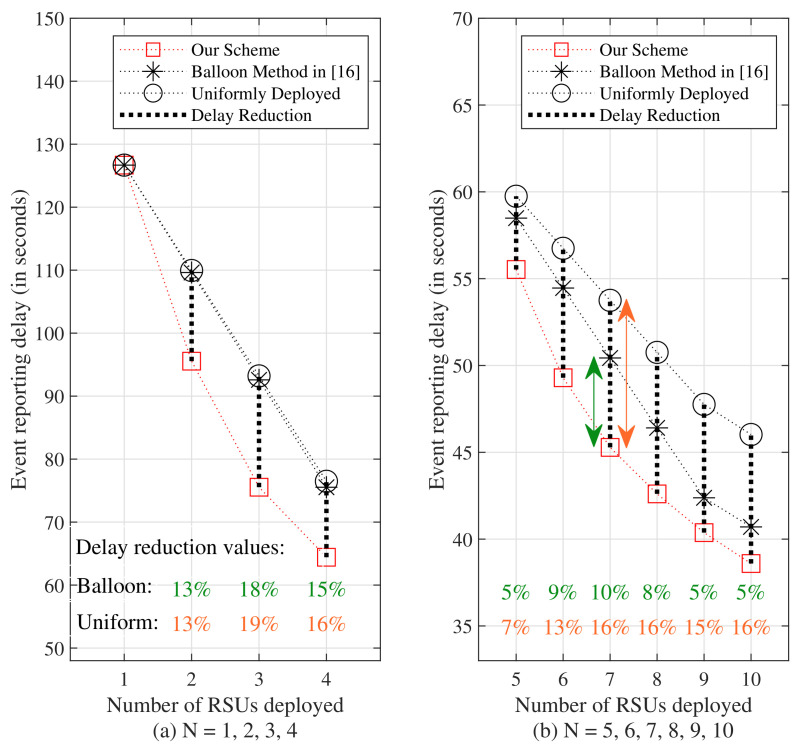
Comparison of event reporting delays of the uniformly distributed scheme, the balloon method in [16], and our scheme, when conducted under the assumption of events occurring with uniform distributions along the road segments, and in (**a**) N=1,2,3,4, in (**b**) N=5,6,7,8,9,10.

Figure 8 illustrates the comparison of event reporting delays of the uniformly distributed scheme, the balloon method in [16], and our scheme for N=1,2,⋯,10. The corresponding delay values are listed in Table 2. It is observed that the delay reduction values range from 7% to 19% when compared with the event reporting delays of the uniformly distributed scheme, and from 5% to 18% when compared with the balloon method, for N>1. Our scheme demonstrates improved performance compared to the other two schemes.

## 7. Conclusions

This paper addresses the intricate task of RSU deployment at highway intersections, a pivotal component of intelligent transportation systems. It offers comprehensive analytical insights into event reporting delays concerning both highway road segments and intersections outfitted with multiple RSUs. In response to the complexities of intersection environments, the paper introduces a novel delay-oriented RSU deployment strategy specifically tailored for highway intersections. Central to this strategy is the cooperative walking algorithm, devised to optimize RSU placement while mitigating event reporting delays effectively. Through illustrative examples, our study demonstrates the superior event reporting delay performance of our proposed approach compared to the traditional uniform RSU deployment method and the balloon method introduced in [16]. For N>1, assuming events occur with truncated normal distributions along the road segments, we observed reductions in delay ranging from 38% to 56% compared to the uniformly distributed scheme, and from 3% to 40% compared to the balloon method. Additionally, when events follow uniform distributions along the road segments, our scheme shows delay reductions ranging from 7% to 19% compared to the uniformly distributed scheme, and from 5% to 18% compared to the balloon method, for N>1. Overall, the findings presented in this paper not only contribute to advancing the understanding of RSU deployment dynamics in complex intersection scenarios but also offer practical insights that can inform the design and implementation of more efficient and reliable intelligent transportation systems.

## Figures and Tables

**Table 1:** Event reporting delays for the uniformly distributed scheme, the balloon method in [16], and our scheme are compared for various values of *N* ranging from 1 to 10, considering events occurring with truncated normal distributions along the road segments.

Number of RSUs	The Uniformly Distributed Scheme	The Balloon Method in [16]	Our Scheme
N=1	204.5839 s	204.5839 s	204.5839 s
N=2	175.8847 s	165.3088 s	108.7988 s
N=3	147.1855 s	126.0338 s	76.6001 s
N=4	118.4862 s	86.7587 s	51.7227 s
N=5	89.7870 s	47.4837 s	45.5996 s
N=6	81.8327 s	44.5893 s	41.3413 s
N=7	73.8784 s	41.6949 s	38.1785 s
N=8	65.9241 s	38.8005 s	36.4358 s
N=9	57.9699 s	35.9061 s	34.8098 s
N=10	54.8607 s	34.6934 s	33.4327 s

**Table 2:** Event reporting delays for the uniformly distributed scheme, the balloon method in [16], and our scheme are compared for various values of *N* ranging from 1 to 10, considering events occurring with uniform distributions along the road segments.

Number of RSUs	The Uniformly Distributed Scheme	The Balloon Method in [16]	Our Scheme
N=1	126.6758 s	126.6758 s	126.6758 s
N=2	109.9437 s	109.6286 s	95.5510 s
N=3	93.2116 s	92.5814 s	75.5394 s
N=4	76.4794 s	75.5342 s	64.4191 s
N=5	59.7473 s	58.4870 s	55.5272 s
N=6	56.7496 s	54.4600 s	49.2931 s
N=7	53.7518 s	50.4330 s	45.2828 s
N=8	50.7541 s	46.4060 s	42.6045 s
N=9	47.7563 s	42.3790 s	40.3740 s
N=10	46.0242 s	40.7028 s	38.5936 s

## Data Availability

No new data were created or analyzed in this study. Data sharing is not applicable to this article.

## Abbreviations

The following abbreviations are used in this manuscript:

ITS	Intelligent transportation systems
RSU	Roadside unit
VANET	Vehicular ad hoc network
PDF	Probability density function
CMP	Capacity maximization placement
RS1	Road Segment 1
RS2	Road Segment 2
RS3	Road Segment 3
RS4	Road Segment 4
CDF	Cumulative distribution function
DS	Double-ended subsegment
SS	Single-ended subsegment
int	Intersection
